# Identification of molecular mechanisms underlying the therapeutic effects of Xintong granule in coronary artery disease by a network pharmacology and molecular docking approach

**DOI:** 10.1097/MD.0000000000029829

**Published:** 2022-07-08

**Authors:** Zhihong Huang, Siyu Guo, Changgeng Fu, Wei Zhou, Antony Stalin, Jingyuan Zhang, Xinkui Liu, Shanshan Jia, Chao Wu, Shan Lu, Bingbing Li, Zhishan Wu, Yingying Tan, Xiaotian Fan, Guoliang Cheng, Yanfang Mou, Jiarui Wu

**Affiliations:** a Department of Clinical Pharmacology of Traditional Chinese Medicine, School of Chinese Materia Medica, Beijing University of Chinese Medicine, Beijing, China; b Xiyuan Hospital of China Academy of Chinese Medical Sciences, Beijing, China; c China-Japan Friendship Hospital, Beijing, China; d Institute of Fundamental and Frontier Sciences, University of Electronic Science and Technology of China, Chengdu, China; e State Key Laboratory of Generic Manufacture Technology of Chinese Traditional Medicine, Shandong Lunan Pharmaceutical Group Co. Ltd., Linyi, China; f College of Chinese Medicine, Beijing University of Chinese Medicine, Beijing, China.

**Keywords:** coronary artery disease, molecular mechanisms, network pharmacology, molecular docking, Xintong granule

## Abstract

Coronary artery disease (CAD) is a cardiovascular disease characterized by atherosclerosis, angiogenesis, thrombogenesis, inflammation, etc. Xintong granule (XTG) is considered a practical therapeutic strategy in China for CAD. Although its therapeutic role in CAD has been reported, the molecular mechanisms of XTG in CAD have not yet been explored.

A network pharmacology approach including drug-likeness (DL) evaluation, oral bioavailability (OB) prediction, protein-protein interaction (PPI) network construction and analysis, and Gene Ontology term and Kyoto Encyclopedia of Genes and Genomes (KEGG) pathway analyses was used to predict the active ingredients, potential targets, and molecular mechanisms of XTG associated with the treatment of CAD. Molecular docking analysis was performed to investigate the interactions between the active compounds and the underlying targets.

Fifty-one active ingredients of XTG and 294 CAD-related targets were screened for analysis. Gene Ontology enrichment analysis showed that the therapeutic targets of XTG in CAD are mainly involved in blood circulation and vascular regulation. KEGG pathway analysis indicated that XTG intervenes in CAD mainly through the regulation of fluid shear stress and atherosclerosis, the AGE-RAGE signaling pathway in diabetic complications, and the relaxin signaling pathway. Molecular docking analysis showed that each key active ingredient (quercetin, luteolin, kaempferol, stigmasterol, resveratrol, fisetin, gamma-sitosterol, and beta-sitosterol) of XTG can bind to the core targets of CAD (AKT1, JUN, RELA, MAPK8, NFKB1, EDN1, and NOS3).

The present study revealed the CAD treatment-related active ingredients, underlying targets, and potential molecular mechanisms of XTG acting by regulating fluid shear stress and atherosclerosis, AGE-RAGE signaling pathway in diabetic complications, and relaxin signaling pathway.

## 1. Introduction

Coronary artery disease (CAD) is a cardiovascular disease that leads to myocardial ischemia, hypoxia, or necrosis due to stenosis or occlusion of the coronary arteries. CAD may be quite extensive and include inflammation, atherosclerosis, angiogenesis, thrombogenesis, apoptosis, and other causes of luminal stenosis or occlusion.^[1–5]^ According to the statistics of the American Heart Association, CAD is the leading cause of death in both developed and developing countries and represents a significant health, economic, and emotional burden worldwide.^[6]^ Some studies have shown that hypertension, dyslipidemia, diabetes, and an unhealthy lifestyle are various risk factors for CAD.^[7–10]^ The immutable risk factors for CAD include age, sex, and family history.^[11–13]^ The diagnosis of CAD depends mainly on typical clinical symptoms, combined with auxiliary examinations to find evidence of myocardial ischemia or coronary artery occlusion and evaluation of myocardial injury markers to determine whether myocardial necrosis has occurred.^[14]^ Currently, the recommended preventive and therapeutic approaches for CAD include lipid-lowering, antithrombotic, and antiinflammatory therapies, percutaneous coronary intervention (PCI), and coronary artery bypass graft (CABG).^[15,16]^ Angiotensin-converting enzyme inhibitors, angiotensin receptor blockers (ARBs), beta-blockers, calcium channel blockers (CCBs), ranolazine, aspirin, P2Y12 receptor antagonists, protease-activated receptor-1 antagonists, and nitrates are recommended for clinical drug therapy.^[17,18]^ Moreover, due to complex pathogenesis, no single treatment can cure CAD. Therefore, multiple intervention approaches should be employed for joint intervention with the pathological process and new strategies are needed to complement existing interventions.

**Figure 1.**
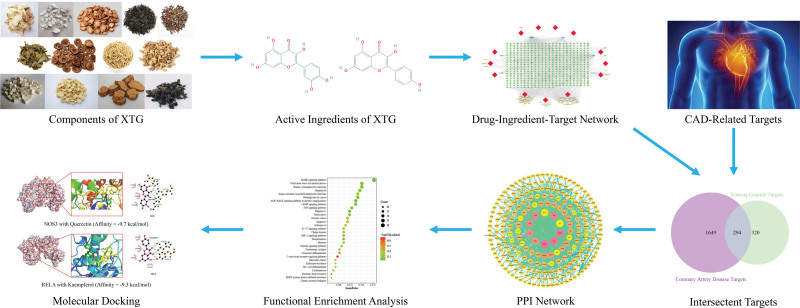
Flowchart summarizing the work scheme of the study.

Traditional Chinese medicine (TCM) relies on TCM theory to collect, process, and prepare the components that can be used to prevent, treat, and diagnose diseases, explain the mechanism of action, and guide clinical application.^[19]^ TCM also provides an effective treatment for CAD. A study by Li showed that TCM could treat CAD via its antiinflammatory effects.^[20]^ Guanxin V is widely used for the clinical treatment of CAD and has been shown to be effective and safe.^[21]^ From the perspective of TCM, the potential for CAD-related therapeutics and drug discovery still need to be improved.^[22]^ Xintong granule (XTG) is widely used to treat CAD in China. XTG contains Huangqi (*Radix Astragalus membranaceus*, HQ), Danshen (*Radix Salvia miltiorrhiza*, DS1), Dangshen (*Radix Codonopsis pilosula*, DS2), Maidong (*Radix Ophiopogon japonicus*, MD), Heshouwu (*Radix Polygonum multiflorum*, HSW), Yinyanghuo (*Epimedium brevicornu*, YYH), Gegen (*Radix Pueraria lobata*, GG), Danggui (*Radix Angelica sinensis*, DG), Zaojiaoci (*Gleditsia sinensis*, ZJC), Haizao (*Sargassum pallidum*, HZ), Kunbu (*Ecklonia kurome*, KB), Muli (*Ostrea gigas*, ML), and Zhishi (*Citrus aurantium*, ZS). Animal experiments have shown that XTG exerts a protective effect on myocardial infarction in rats and a hypolipidemic effect. However, the molecular mechanisms of XTG against CAD remain unclear. In this study, a network pharmacology strategy was employed to explore the multiple components, targets, and signaling pathways closely associated with the therapeutic effect of XTG in CAD. A molecular docking approach was applied to verify the binding of the active ingredients to the action targets to reveal and predict the efficacy of XTG for the treatment of CAD. The detailed workflow is shown in Figure 1.

## 2. Methods

### 2.1. Screening of active ingredients and predicted targets of XTG and construction of the network

All chemical constituents of XTG were retrieved from the Traditional Chinese Medicine Systems Pharmacology Database and Analysis Platform (TCMSP, https://tcmsp-e.com/) and the Bioinformatics Analysis Tool for Molecular mechANism of Traditional Chinese Medicine (BATMAN-TCM, http://bionet.ncpsb.org.cn/batman-tcm/). These databases, which contain the relationships between drugs, targets, and diseases, are the most commonly used databases for searching the components of TCMs.^[23,24]^ The absorption, distribution, metabolism, and excretion (ADME) parameters “oral bioavailability (OB) ≥ 30%, drug-likeness (DL) ≥ 0.18” and “validated” status were set as filtering criteria for the active ingredients and their predicted targets in the TCMSP database. Some XTG components could not be retrieved from the TCMSP database and were therefore searched in the BATMAN-TCM database. The active compounds of XTG and their predicted targets were obtained according to the input parameter “score cutoff ≥ 20”. The predicted targets were further standardized using the UniProt database (https://www.uniprot.org/) and corrected to the official gene names.^[25]^ Cytoscape 3.7.0 software was used to construct the drug-ingredient-target network of XTG.^[26]^

### 2.2. Collection of CAD-related targets

The CAD-related targets were collected by integrating data from the following well-recognized disease databases: (1) Therapeutic Target Database (TTD, http://db.idrblab.net/ttd/),^[27]^ (2) Comparative Toxicogenomics Database (CTD, http://ctdbase.org/),^[28]^ (3) DrugBank (https://drugbank.ca/),^[29]^ and (4) GeneCards (https://www.genecards.org/).^[30]^ The keyword “coronary artery disease” and the species “*Homo sapiens*” were set as screening parameters.

### 2.3. Identification of potential therapeutic targets of XTG in CAD

Venn diagrams are commonly used to show the similarities and differences between gene lists in biological contexts.^[31]^ Through Venn diagram construction, the obtained XTG targets were intersected with the CAD-related targets, and the intersecting targets were considered as potential therapeutic targets of XTG in CAD.

### 2.4. PPI network construction and analysis

The Search Tool for the Retrieval of Interaction Genes/Proteins (STRING, https://string-db.org/) database is widely used to predict protein-protein interactions (PPIs).^[32]^ The common targets of XTG and CAD were imported into the STRING database, and the minimum required interaction score was chosen as “high confidence (0.700)” and the species was set as “*Homo sapiens*” to acquire the interaction relationships among the targets. The data were then imported into Cytoscape 3.7.0 software to construct the PPI network. NetworkAnalyzer was used to calculate the topological parameters of the PPI network, such as node degree, betweenness centrality, and closeness centrality. According to the topological relationships, the targets that met the criteria of degree ≥ average value (8.98), betweenness centrality ≥ average value (0.0117), and closeness centrality ≥ average value (0.266) were considered as core target candidates.

### 2.5. Protein functional enrichment analysis

Gene Ontology (GO) is an international standard system for functional classification of genes.^[33]^ Kyoto Encyclopedia of Genes and Genomes (KEGG) is a knowledge base for systematic analysis of gene functions that links genomic information with pathway annotations.^[34]^ GO and KEGG functional enrichment analyses are widely applied to reveal the potential biological functions of genes. The Database for Annotation, Visualization, and Integrated Discovery (DAVID, https://david.ncifcrf.gov/) was adopted to predict the molecular mechanisms of XTG in CAD treatment.^[35]^ The results of GO biological process (BP) and KEGG pathway enrichment analysis were saved and sorted by false discovery rate (FDR)-corrected and adjusted *P* values for each term.

### 2.6. Screening of key active ingredients and core targets

After analyzing the drug-ingredient-target network of XTG, the key active ingredients were identified according to the topological parameter degree ≥ average value (32.32) and annotation of their use for the treatment of CAD in the CTD. The core targets were obtained by intersecting the targets in the BPs and pathways identified by GO and KEGG functional enrichment analysis as highly correlated with CAD.

### 2.7. Verification of key active ingredients and core targets by molecular docking analysis

Molecular docking analysis was performed to validate whether the key active ingredients could bind to the core targets.^[36]^ First, the 2D structures of the key active ingredients were downloaded from the PubChem database (https://pubchem.ncbi.nlm.nih.gov/).^[37]^ The structures were imported into AutoDockTools-1.5.6 software to add charges and display rotatable bonds, and were then saved in pdbqt format. Second, the crystal structures of the proteins corresponding to the core targets were downloaded from the Protein Data Bank (PDB, https://www.rcsb.org/).^[38]^ Then, these structures were imported into PyMOL software to remove water molecules and heteromolecules, and imported into AutoDockTools-1.5.6 to add hydrogen atoms and charges and saved in pdbqt format. Finally, the 3D grid box for molecular docking simulation was created using AutoDockTools-1.5.6 and visualized with AutoDock Vina 1.1.2.^[39]^ The results were analyzed and visualized using PyMOL and Ligplot software.

## 3. Results

### 3.1. Drug-ingredient-target network construction

**Figure 2.**
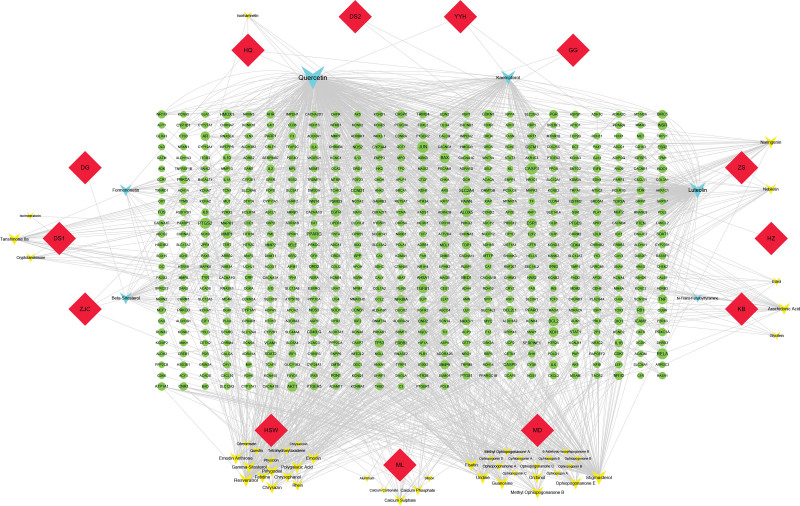
Drug-ingredient-target network of XTG. Red diamonds represent the drugs of XTG; yellow Vs represent active ingredients in each drug; blue Vs represent active ingredients shared by multiple drugs, and green ellipses correspond to related targets. The larger the node, the greater the degree value, and the closer the prompt relationship.

In the present study, 53 active ingredients and 614 predicted targets were identified from the thirteen drugs in XTG, as shown in Supplementary Table S1, http://links.lww.com/MD/G838. The drug-ingredient-target network revealed complex ingredients, multiple targets, and close interactions between ingredients and targets, as shown in Figure 2. According to the topological parameters degree ≥ average value (32.32) and annotation on the use for the treatment of CAD in the CTD, the 8 key active ingredients that may play an important role in the therapeutic effect of XTG in CAD were identified as quercetin, luteolin, kaempferol, stigmasterol, resveratrol, fisetin, gamma-sitosterol, and beta-sitosterol, as shown in Table 1.

**Table 1:**
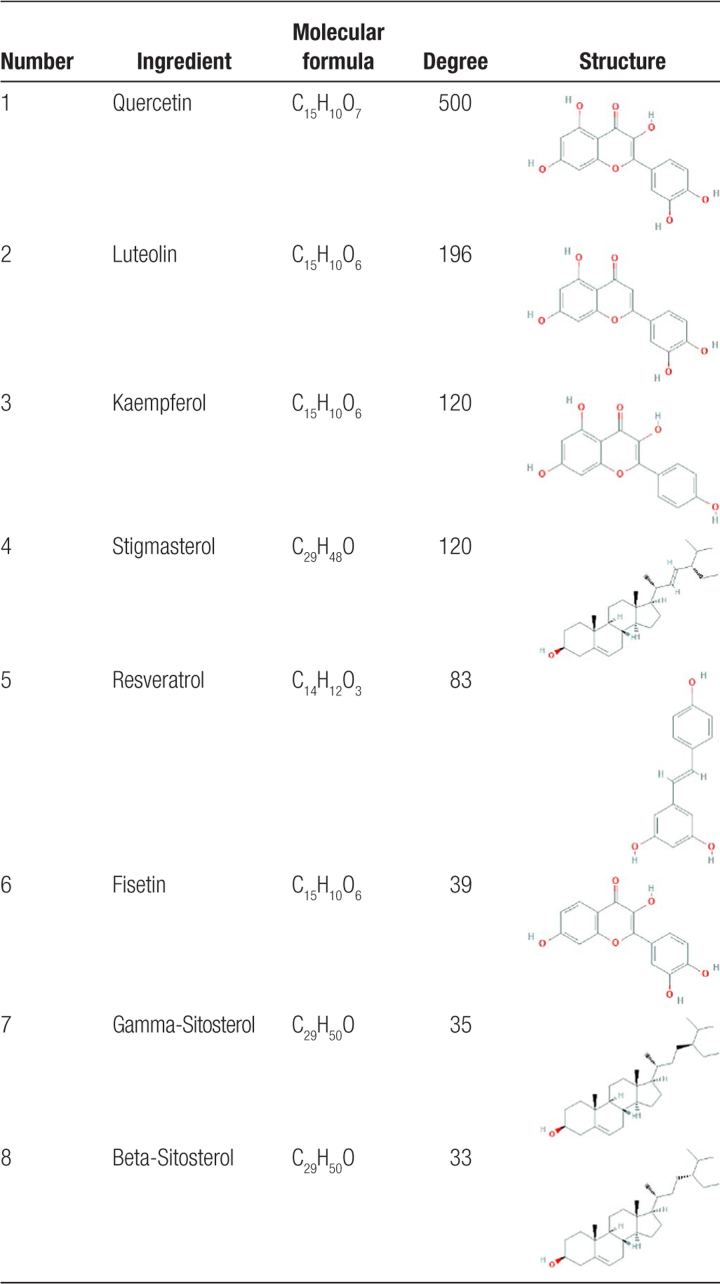
Key active ingredients of XTG.

### 3.2. Potential therapeutic targets of XTG in CAD

**Figure 3.**
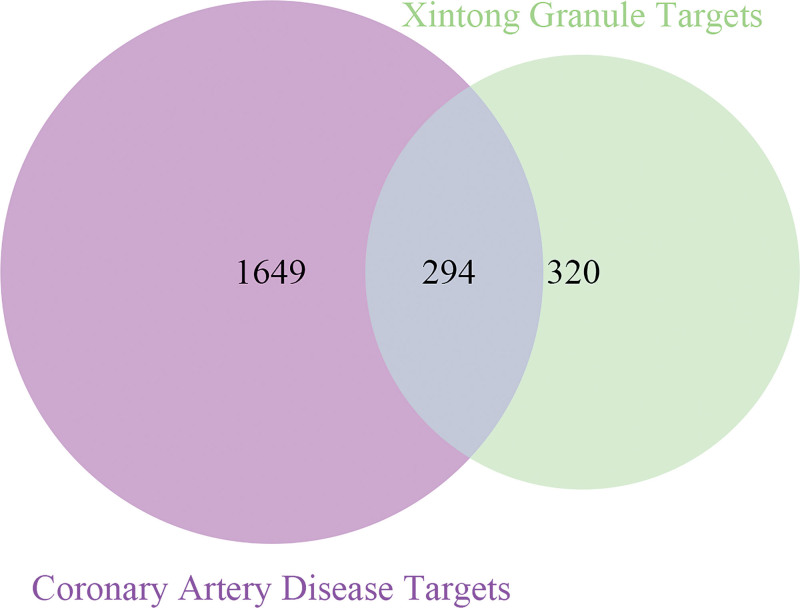
Venn diagram of XTG and CAD targets.

The CAD-related targets were integrated from the multisource database, and 1943 disease-related targets were obtained after the elimination of duplicates, as shown in Supplementary Table S2, http://links.lww.com/MD/G839. The targets of XTG and CAD were used to construct a Venn diagram for mapping and intersection. A total of 294 overlapping targets associated with 51 active ingredients were considered as potential therapeutic targets of XTG in CAD, as shown in Figure 3 and Supplementary Table S3, http://links.lww.com/MD/G840.

### 3.3. PPI network construction

**Figure 4.**
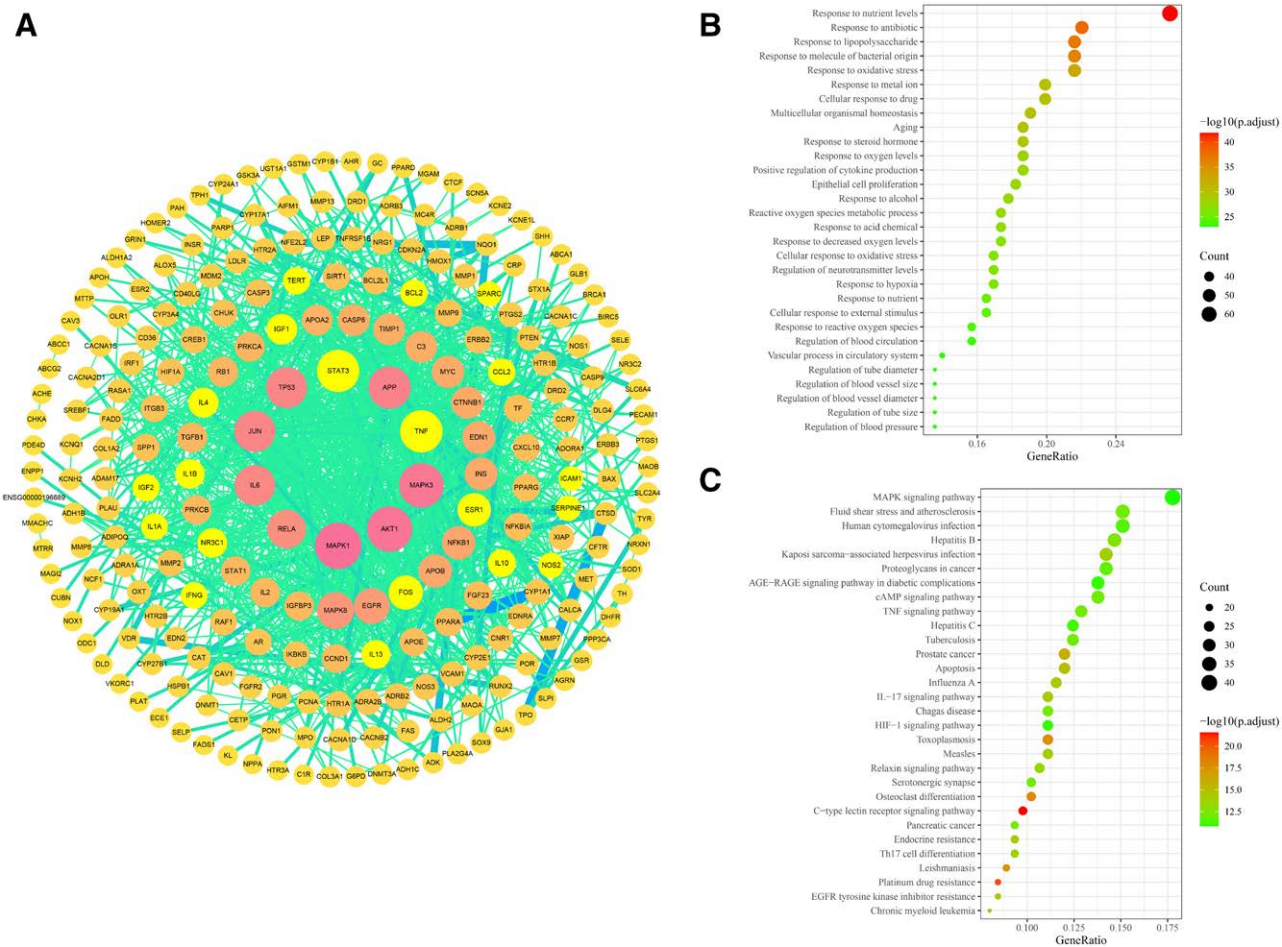
(A) PPI network of XTG for CAD treatment. Each node represents a protein target and each line represents the interaction between 2 nodes. The color of the node from yellow to pink and the size of the node from small to large, the degree was increasing. The color of the edge from green to blue and the width of the edge from thin to thick, the interaction strength become stronger. (B) The top 30 results of GO-BPs term enrichment analysis of targets. (C) The top 30 results of KEGG pathways enrichment analysis of targets.

The 294 intersected targets were used to construct the PPI network using the STRING database and Cytoscape 3.7.0 software. The network contained 237 nodes and 1064 edges, as shown in Figure 4A. After PPI network analysis, 34 core candidate targets that met the criteria of degree ≥ average value (8.98), betweenness centrality ≥ average value (0.0117), and closeness centrality ≥ average value (0.266) were selected according to the topological parameters, as shown in Supplementary Table S4, http://links.lww.com/MD/G841.

### 3.4. GO and KEGG functional enrichment analyses

Through GO BP analysis, the top 30 BPs of potential therapeutic targets of XTG were identified in CAD. The targets were mainly enriched in the terms of cellular responses to stimuli, regulation of biological quality, metabolism, blood circulation, and vascular regulation. From the data in Figure 4B and Supplementary Table S5, http://links.lww.com/MD/G842, it can be speculated that the therapeutic effect of XTG in CAD may be correlated primarily with the following BP terms: (1) regulation of blood circulation, (2) vascular process in the circulatory system, (3) regulation of blood vessel size, (4) regulation of blood vessel diameter, and (5) regulation of blood pressure.

To further explore the potential molecular mechanisms of XTG in CAD treatment, the top 30 signaling pathways were examined in detail, as shown in Figure 4C and Supplementary Table S6, http://links.lww.com/MD/G843. The biological effects of these signaling pathways suggest that the key active ingredients of XTG exert their effects on CAD via regulating the following signaling pathways: (1) fluid shear stress and atherosclerosis, (2) AGE-RAGE signaling pathway in diabetic complications, and (3) relaxin signaling pathway. Based on the BPs and signaling pathways identified above, AKT1, JUN, RELA, MAPK8, NFKB1, EDN1, and NOS3 were considered as the core targets, as shown in Table 2.

**Table 2:** Core targets of XTG in the treatment of CAD.

Number	Target name	Degree	Betweenness centrality	Closeness centrality
1	AKT1	44	0.096075	0.442913
2	JUN	37	0.056292	0.443787
3	RELA	33	0.026494	0.43021
4	MAPK8	30	0.027659	0.422932
5	NFKB1	24	0.037336	0.410584
6	EDN1	23	0.050285	0.411335
7	NOS3	11	0.011983	0.371901

### 3.5. Verification of key active ingredients and core targets by molecular docking analysis

**Figure 5.**
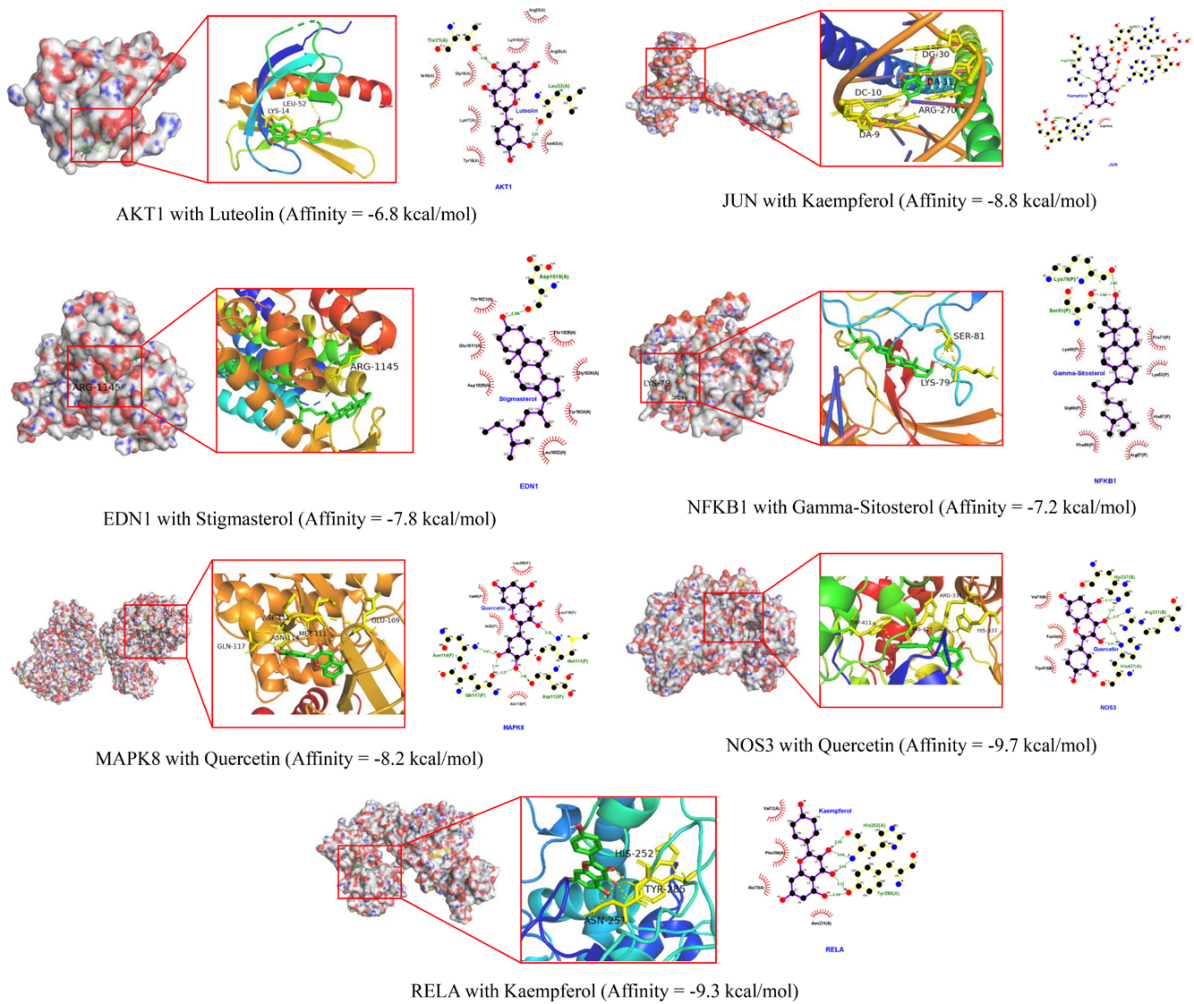
Molecular docking diagram of core targets complexed with key active ingredients.

The results obtained by molecular docking software are shown in Table 3. The grid box was centered on covering the active binding site and all essential residues. For AKT1, the grid box (46 Å × 38Å × 54Å) was centered at (12.538, −0.667, −2.487) Å; for JUN, the grid box (78 Å × 78Å × 78Å) was centered at (−14.083, 17.354, 22.233) Å; for RELA, the grid box (88 Å × 88Å × 88Å) was centered at (62.86, 8.212, 40.649) Å; for MAPK8, the grid box (88 Å × 104Å × 126Å) was centered at (−48.256, 15.86, 65.048) Å; for NFKB1, the grid box (70 Å × 70Å × 80Å) was centered at (38.959, 11.032, 32.044) Å; for EDN1, the grid box (58 Å × 86Å × 68Å) was centered at (19.158, 30.479, 3.206) Å; and for NOS3, the grid box (76 Å × 72Å × 80Å) was centered at (17.922, 12.101, 47.548) Å. The binding affinities of the key active ingredients for the protein crystal structures corresponding to the core targets were all greater than −5 kcal/mol, indicating that all ingredients had an affinity for all protein crystal structures. Figure 5 shows that the small molecule ingredients were tightly bound to the protein residues via various interactions.

**Table 3:**
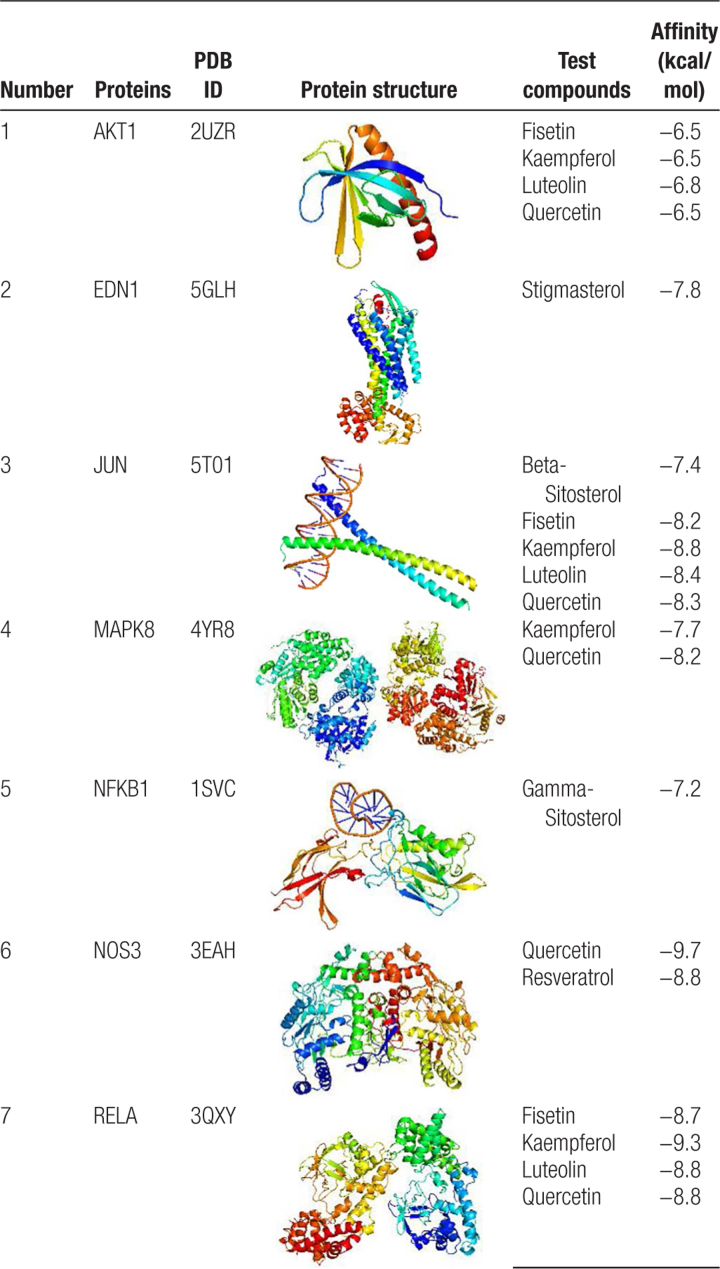
The molecular docking results of key active ingredients and core targets.

## 4. Discussion

CAD is a major public health challenge in China, and some scholars have written treatment guidelines to address this situation.^[40]^ Currently, CAD is mainly treated with antiinflammatory, antiapoptotic, antiatherosclerotic, antiangiogenic, and antithrombogenic agents. TCM has a long history in clinical practice and is widely used for the treatment of diseases; in addition, many resources are worthy of further study.^[41]^ Among the complex ingredients in herbal medicines, unique combinations in traditional complex formulas may play an important role in intervention with specific chronic diseases by targeting specific cell signaling nodes.^[42]^ Network pharmacology has recently been used to explore the intervention mechanisms of drugs in diseases. It systematically reveals the therapeutic effects of drugs in diseases based on the interactions between drugs, targets, and diseases.^[43,44]^ To better understand the potential mechanisms of XTG, network pharmacology and molecular docking analyses were used to determine the underlying molecular mechanisms of its bioactive compounds.

Network topology analysis revealed that the key active ingredients in XTG are quercetin, luteolin, kaempferol, stigmasterol, resveratrol, fisetin, gamma-sitosterol, and beta-sitosterol. These compounds are reported to have antiinflammatory, hypolipidemic, and cardioprotective effects, indicating their potential use in the treatment of CAD.^[45–50]^ Moreover, quercetin can reduce the transcriptional activity of nuclear factor kappa B (NF-κB), thereby decreasing the levels of inflammatory cytokines such as IL-1β, IL-10, and TNF-α, which may treat CAD via antiinflammatory effects.^[51]^ Resveratrol increases nitric oxide (NO) production in endothelial cells, stimulates endothelial nitric oxide synthase (eNOS) activity, and prevents eNOS uncoupling by upregulating its expression. In addition, resveratrol inhibits the synthesis of endothelin-1 (ET-1) and reduces oxidative stress in endothelial cells and smooth muscle cells. Resveratrol can also improve pathological stimulation-induced smooth muscle cell proliferation, vascular remodeling, and arterial stiffness, thus preventing and treating arterial development of CAD.^[49]^ These previous findings suggest that XTG probably decrease the levels of inflammatory cytokines, inhibits the proliferation of vascular endothelial cells, and prevents thrombogenesis and atherosclerosis in CAD mainly by quercetin, luteolin, kaempferol, stigmasterol, resveratrol, fisetin, gamma-sitosterol, and beta-sitosterol.

Our results suggest that the core target genes of XTG are enriched primarily in the following signaling pathways: fluid shear stress and atherosclerosis, AGE-RAGE signaling pathway in diabetic complications, and relaxin signaling pathway. These signaling pathways are related to inflammation, angiogenesis, atherogenesis, vasodilation and thrombogenesis. In the endothelium, AKT1 is the main AKT subtype and phosphorylates eNOS directly at Ser1177.^[52]^ NO is the main endothelium-derived regulator of vascular tension.^[53]^ Deactivation of AKT1, leading to a decrease in phosphorylation of eNOS (encoded by NOS3), results in a decrease in NO.^[54]^ A decrease in NO causes proliferation of vascular smooth muscle cells (VSMCs), resulting in vascular proliferative diseases, is a pathophysiological mechanism of CAD.^[55]^ AKT1 can translocate to the nucleus, bind to RELA to form a complex, and activate the transcription of NF-κB.^[56]^ Some researchers found that macrophage infiltration and expression of proinflammatory genes (TNF-α and IL-6) were increased in vessels lacking AKT1. TNF-α and IL-6 are highly expressed by foam cell macrophages, and increased expression of VCAM-1 and ICAM-1 in endothelial cells supports further recruitment of monocytes into atherosclerotic plaques.^[57]^ Increased expression of NOS3 inhibits caspase-3 (CASP3) expression and activity, preventing cardiomyocyte apoptosis.^[58]^ MAPK8 (JNK1), a member of the mitogen-activated protein kinase family of serine-threonine protein kinases, is a positive regulator of c-Jun expression, cell proliferation, and vascular physiology.^[59]^ Activation of JNK1 induces NF-κB activation and upregulates TNF-α and IL-6 expression, leading to inflammation and atherosclerosis.^[60,61]^ In addition, JNK1 mediates dissociation of the Bcl-2/Beclin 1 complex, leading to apoptosis.^[62]^ NF-κB (composed of RELA and NFKB1) is a transcriptional regulator of inflammation-related genes and plays an important role in atherosclerosis.^[63]^ Some studies have shown that NF-κB selectively upregulates proinflammatory and prothrombotic mediators in atherosclerosis.^[64,65]^ Researchers found that downregulation of NF-κB with specific NF-κB inhibitors can reduce inflammation and prevent atherosclerosis.^[66,67]^ Vascular endothelial growth factor is a proangiogenic factor in pathological processes, and NF-κB activation is positively correlated with the Vascular endothelial growth factor production.^[68,69]^ Activating protein-1 (AP-1) is a collective term for dimeric transcription factors consisting of the subunits JUN, FOS, and ATF that are positively regulated by JNKs.^[70]^ After AP-1 is activated, the JNK protein translocates to the nucleus and phosphorylates c-Jun and activating transcription factor-2. As a results, the level of inflammatory cytokines increases, leading to inflammation.^[71,72]^ ET-1 is a potent vasoconstrictor peptide linked to vascular diseases through EDN1. ET-1 mediates the activation of the G protein-coupled receptor endothelin A (ETA) in VSMCs, leading to endothelial dysfunction, inflammation, atherosclerosis, and vascular proliferation.^[73]^ A study by Liang showed that the EDN1 tag SNP rs6458155 was associated with CAD risk in the Chinese Han population, possibly due to an increase in circulating ET-1 level.^[74]^

**Figure 6.**
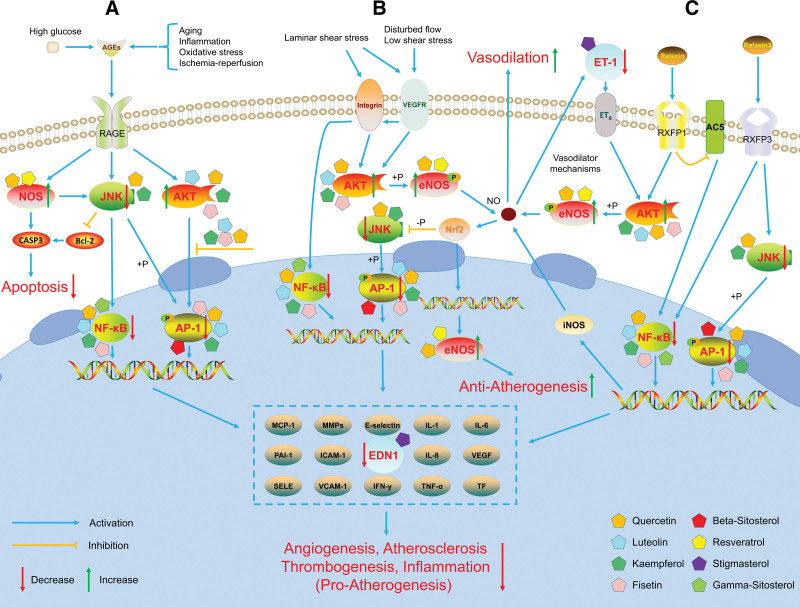
Hub molecular mechanisms of XTG in the treatment of CAD. (A) AGE-RAGE signaling pathway in diabetic complications. (B) Fluid shear stress and atherosclerosis. (C) Relaxin signaling pathway. The targets of red font are the core targets.

By summarizing the results of previous studies and the present study, we hypothesized the molecular mechanisms of XTG in the treatment of CAD, as shown in Figure 6. (1) Fluid shear stress and atherosclerosis: XTG upregulates the expression of AKT1 and NOS3, directly phosphorylates eNOS, and increases the production of NO, resulting in vasodilation and antiinflammatory and antithrombotic effects, thereby ameliorating atherosclerosis. XTG can also downregulate the expression of MAPK8, RELA, NFKB1, JUN, and EDN1, and inhibit JNK-induced activation of NF-κB and AP-1. It can treat CAD by reducing the production of inflammatory cytokines and ET-1, which may cause inflammation, angiogenesis, and VSMC proliferation and migration. (2) AGE-RAGE signaling pathway in diabetic complications: XTG inhibits the expression and activity of CASP3 by upregulating the expression of NOS3 and downregulating the expression of MAPK8, thereby preventing apoptosis. XTG downregulates the expression of MAPK8, RELA, NFKB1, JUN, and EDN1, inhibits the nuclear translocation of AKT1, and the binding of AKT1 to RELA to activate NF-κB. These effects, in turn, suppress the activity of AP-1 and NF-κB and reduce the production of inflammatory cytokines, growth factors, and adhesion molecules that induce inflammation, thrombogenesis, atherosclerosis and angiogenesis. (3) Relaxin signaling pathway: XTG increases the expression of AKT1 and NOS3 and activates eNOS to produce NO, resulting in vasodilation. XTG downregulates the expression of MAPK8, RELA, NFKB1, JUN, and EDN1, decreases the levels of NF-κB and AP-1, and reduces the transcription of inflammatory cytokines, growth factors, and adhesion molecules.

## 5. Conclusions

As described above, a network pharmacology strategy was proposed to investigate the CAD treatment-related active ingredients, potential therapeutic targets, and regulatory pathways of XTG. The results revealed the active compounds of XTG and the underlying molecular mechanisms by which XTG protects against inflammation, thrombogenesis, atherosclerosis, and angiogenesis by regulating fluid shear stress and atherosclerosis, AGE-RAGE signaling pathway in diabetic complications and relaxin signaling pathway. These findings were partially verified by previous studies. Molecular docking analysis showed that each key active ingredient (quercetin, luteolin, kaempferol, stigmasterol, resveratrol, fisetin, gamma-sitosterol, and beta-sitosterol) of XTG had a favorable binding affinity for the core target (AKT1, JUN, RELA, MAPK8, NFKB1, EDN1, and NOS3), further supporting the potential molecular mechanisms of XTG in CAD. This study provides a comprehensive reference for investigating the therapeutic mechanisms of XTG in CAD.

### Author contributions

Conceptualization: Jiarui Wu.

Data curation: Shan Lu, Zhishan Wu, Yingying Tan, Xiaotian Fan.

Formal analysis: Chao Wu, Shanshan Jia, Guoliang Cheng, Bingbing Li.

Methodology: Siyu Guo, Changgeng Fu, Wei Zhou, Xinkui Liu, Jingyuan Zhang.

Supervision: Jiarui Wu, Yanfang Mou.

Writing – original draft: Zhihong Huang.

Writing – review & editing & language polishing: Zhihong Huang, Siyu Guo, Antony Stalin, Jiarui Wu.

### Acknowledgments

The authors are thankful to Beijing University of Chinese Medicine, Xiyuan Hospital of China Academy of Chinese Medical Sciences and Shandong Lunan Pharmaceutical Co., Ltd. for the help in conducting this study.

## Supplementary Material


